# Navigating to new frontiers in behavioral neuroscience: traditional neuropsychological tests predict human performance on a rodent-inspired radial-arm maze

**DOI:** 10.3389/fnbeh.2014.00294

**Published:** 2014-09-09

**Authors:** Sarah E. Mennenga, Leslie C. Baxter, Itamar S. Grunfeld, Gene A. Brewer, Leona S. Aiken, Elizabeth B. Engler-Chiurazzi, Bryan W. Camp, Jazmin I. Acosta, B. Blair Braden, Keley R. Schaefer, Julia E. Gerson, Courtney N. Lavery, Candy W. S. Tsang, Lauren T. Hewitt, Melissa L. Kingston, Stephanie V. Koebele, K. Jakob Patten, B. Hunter Ball, Michael K. McBeath, Heather A. Bimonte-Nelson

**Affiliations:** ^1^Department of Psychology, Arizona State University, Tempe, AZ, USA; ^2^Arizona Alzheimer's Consortium, Phoenix, AZ, USA; ^3^Barrow Neurological Institute, St. Joseph's Hospital and Medical Center, Phoenix, AZ, USA

**Keywords:** cognition, working memory, memory, neuropsychological test, rodent, human

## Abstract

We constructed an 11-arm, walk-through, human radial-arm maze (HRAM) as a translational instrument to compare existing methodology in the areas of rodent and human learning and memory research. The HRAM, utilized here, serves as an intermediary test between the classic rat radial-arm maze (RAM) and standard human neuropsychological and cognitive tests. We show that the HRAM is a useful instrument to examine working memory ability, explore the relationships between rodent and human memory and cognition models, and evaluate factors that contribute to human navigational ability. One-hundred-and-fifty-seven participants were tested on the HRAM, and scores were compared to performance on a standard cognitive battery focused on episodic memory, working memory capacity, and visuospatial ability. We found that errors on the HRAM increased as working memory demand became elevated, similar to the pattern typically seen in rodents, and that for this task, performance appears similar to Miller's classic description of a processing-inclusive human working memory capacity of 7 ± 2 items. Regression analysis revealed that measures of working memory capacity and visuospatial ability accounted for a large proportion of variance in HRAM scores, while measures of episodic memory and general intelligence did not serve as significant predictors of HRAM performance. We present the HRAM as a novel instrument for measuring navigational behavior in humans, as is traditionally done in basic science studies evaluating rodent learning and memory, thus providing a useful tool to help connect and translate between human and rodent models of cognitive functioning.

## Introduction

Spatial learning and memory, the ability to encode, store, and retrieve information about route navigation and object locations (Barnes et al., 1997), has been a major focus in the field of neuroscience since Tolman famously asserted that rodents utilize cognitive maps of their environments to navigate mazes (Tolman, 1948). Several decades and many landmark findings later, an abundance of rodent research probing the many facets of spatial navigation and numerous useful tools for measuring spatial learning and memory have been amassed (see Bimonte-Nelson et al., 2010 for review). In rodents, one of the most commonly used and widely recognized tests of spatial memory is the radial-arm maze (RAM) (Olton and Samuelson, 1976; Jarrard, 1993), which consists of a circular arena, from which multiple arms radiate outward. Rewards are typically located at the end of each arm, or a subset of the arms, depending on the specific task protocol, and the maze is surrounded by plentiful extra-maze environmental cues to aid in spatial navigation. The maze relies on positive and/or negative reinforcement to motivate animals to efficiently locate each reward using the fewest arm entries possible.

In the RAM task, rewards are typically not replaced once they have been located within each testing session, resulting in increasing task difficulty (i.e., the number of spatial locations the animal must avoid for successful performance) across trials, within each testing session. In the animal research literature, working memory is considered to be a form of short-term memory and is classically defined as information that is worked with, kept “online,” and updated. In the RAM, working memory demand is elevated with each trial; once a reward is located at the end of an arm, the animal must then remember to avoid that arm on future trials for optimal task performance. This complexity makes the RAM a valuable instrument for evaluating the ability to handle a systematic increase in working memory load. It is well documented in both rats and mice that RAM errors increase within each day as trials progress and working memory demand escalates; however, errors decrease across multiple testing sessions as animals learn the task (Olton and Samuelson, 1976; Jarrard, 1993; Hyde et al., 1998; Bimonte and Denenberg, 1999; Bimonte-Nelson et al., 2003; Camp et al., 2012).

Evidence supports the assertion that, in humans, spatial learning and memory involves multiple complex cognitive processes similar to those measured in rodents. For example, in order to form a cognitive spatial map, humans also acquire knowledge about environmental cues (Taylor and Tversky, 1992; Shelton and McNamara, 2004). Additionally, human neuroimaging studies have discovered cell analogs to rodent place cells in the hippocampus, providing support for brain mechanisms similar to those of rats when mediating navigation through space (Ekstrom et al., 2003), further supporting the idea that humans, like rats, utilize a “cognitive map” of their environment. Many effective tasks have been developed to tap visuospatial ability, episodic memory, and working memory capacity in humans in both experimental and clinical settings. Tasks measuring general intelligence in humans, a domain that has yet to be defined or tested in rodents, have also been widely developed.

Rodent assessments of spatial memory are often also assessments of episodic memory, working memory capacity, as well as visuospatial ability. Rodent models have been critical to our understanding of spatial learning and memory, the brain regions and mechanisms that confer navigational skills, and potential therapies and pharmacological treatments to improve quality of life in populations suffering from cognitive impairments. Rodent RAM research, specifically, has produced a wealth of translational knowledge by allowing for pharmacological, genetic, and environmental manipulations that are not ethically or logistically possible in human populations. Data collected with the rodent RAM have led researchers toward numerous discoveries and new directions with the potential to enrich and optimize cognitive function in humans; use of this paradigm is essential to decipher the infinitely complex neural mechanisms associated with learning and memory, as well as the influence of aging, disease, environmental changes, and countless other factors. It is generally thought that rodent performance on the RAM depends on visuospatial ability, working memory capacity, and an intact episodic memory, but not general intelligence. These same cognitive domains are readily evaluated in humans; however, it remains unclear whether working definitions of these cognitive domains in rodent and human research are functionally equivalent. The extent to which rodent RAM research is directly translational to human learning and memory persists as a key scientific question.

One approach to this immensely complex and dynamic issue is to create an intermediate testing instrument by adapting experimental paradigms from animals to humans. Our aim in the present study was just that—to use a direct and literal translational approach to design a human task that measures the ability to remember and utilize information about spatial locations in a real world, walk-though environment, modeled after rodent RAMs. We assembled a complementary team of scientists with expertise in rodent maze learning, human perception and memory, navigational behavior, and diagnostic clinical neuropsychology. We constructed an 11-arm, walk-through human RAM (HRAM), aiming to make the task as similar as possible to the rodent RAM, and compared performance on the HRAM to performance on a battery of tests tapping cognitive domains that are hypothesized to underlie spatial learning and memory; namely, spatial reasoning ability, episodic memory, working memory, and general intelligence. The HRAM allowed us to translate and compare navigational error patterns, exactly as measured in rodent RAM studies, to performance on a battery of standard neuropsychological and cognitive tests in human participants.

Our primary goal was to determine whether the HRAM produces a similar pattern of errors to that seen in rodents both within and across testing sessions. We expected to see HRAM errors change as a function of WM load and testing session. Specifically, we predicted that HRAM performance would decline as working memory demand became elevated within each testing session, but that performance would improve across testing sessions, similar to the pattern of performance seen in rodents. An additional goal of this study was to explore the relationship between HRAM performance and performance on commonly used neuropsychological and cognitive tests. In order to better understand the relationship between some of the most commonly used rodent and human methodology, we aimed to determine how much variance in HRAM performance could be predicted by scores on standard tests of visuospatial ability, working memory, episodic memory, and general intellectual ability. Because RAM performance relies on working memory and knowledge of spatial locations, we hypothesized that participants' scores on tests (defined in Methods Section) of working memory capacity (OSpan, RSpan, RotSpan, SymSpan), and visuospatial ability (MRT, JLAP) would predict performance on the HRAM. We also tested whether performance on a measure of episodic memory (RAVLT) would predict HRAM performance. We also wanted to investigate whether a measure of general intelligence would predict performance on the HRAM. The final goal of this project was to determine whether tasks that measure different domains of cognition in humans would account for unique portions of variance in HRAM scores, that is, whether each class of tests (i.e., working memory capacity tasks, visuospatial ability tasks, episodic memory tasks) contributed distinctly to overall prediction of HRAM performance. We aimed to assess the extent to which the addition of neuropsychological tests to a standard battery of cognitive tests would improve prediction of human ability to navigate and learn in a real-world environment. We predicted that performance on each group of tasks would account for unique variance in our HRAM task. The overarching goal of this study was to help expand knowledge of both human and rodent cognition, to allow broader interpretations of existing data in both species, and to facilitate translational connections between animal laboratory, human laboratory, and human clinical research domains.

## Materials and methods

### Participants

A total of 157 participants (54 men and 103 women) were recruited from several psychology courses at Arizona State University^1^. Mean age was 21.29 years (SD = 3.75, range = 18–47). Mean educational level in years was 14.43 (SD = 1.29, 13–18 years range). There were no sex differences in age, education, or self-reported college GPA. Participation in the study was an option for extra credit in those courses. All procedures were approved by an Institutional Review Board for use of human participants in research. Names were used only to assign course credit; all performance or questionnaire data were de-identified. All participants had normal or corrected to normal vision and no other obvious physical difficulties with the potential to affect their performance in the maze.

### Human radial arm maze

**Figure 1:**
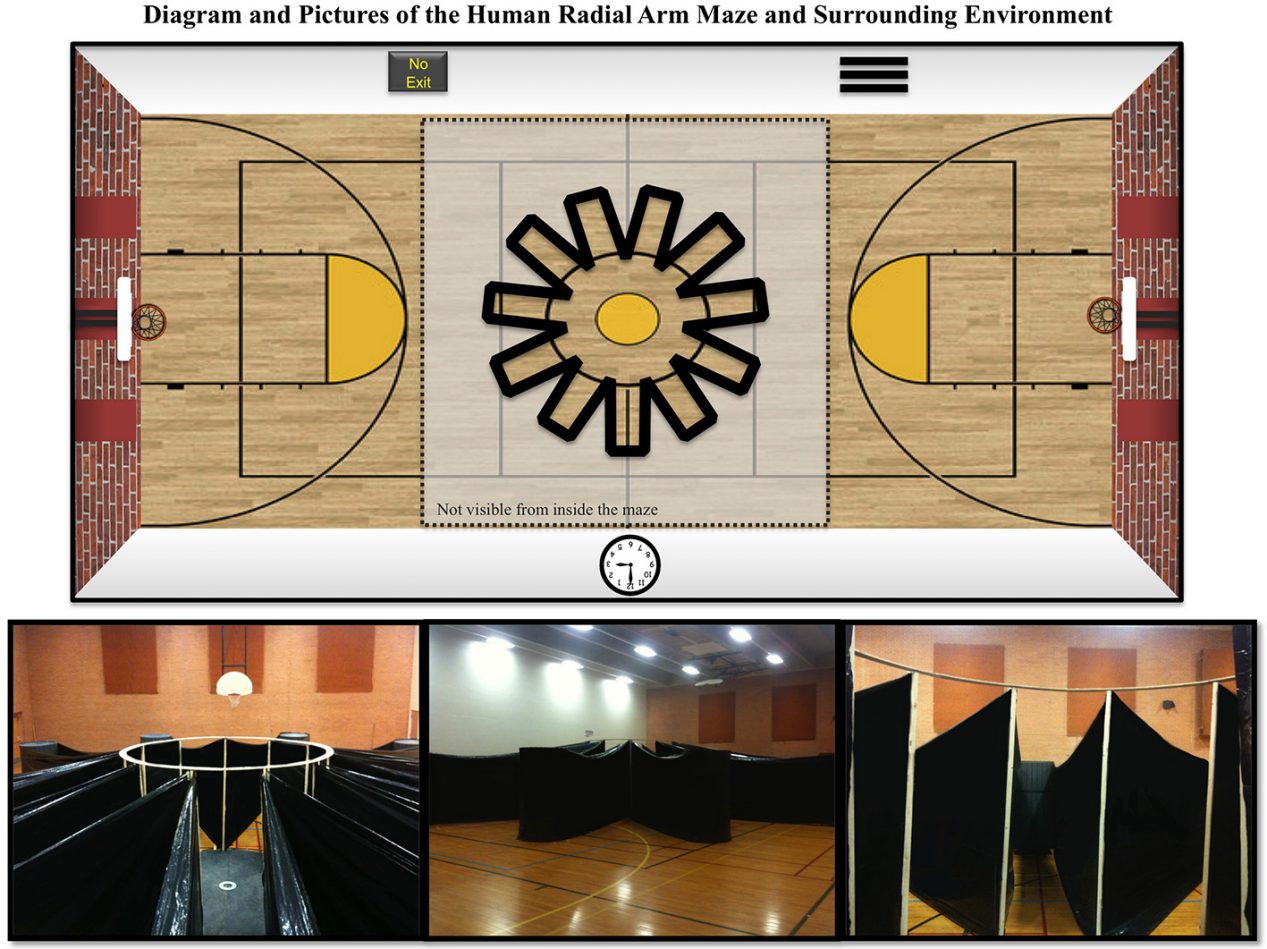
**Schematics and pictures of the Human Radial Arm Maze (HRAM)**. Schematic showing that the HRAM consisted of 11 equally sized arms radiating out from a central circular location. Also presented are: a view from atop the end of one of the arms looking toward the circular center region, an outside view of the HRAM, and a view from the circular center region looking out at several arms with spatial cues in the background.

We developed and constructed a novel HRAM to fit human proportions. A schematic and pictures of the HRAM are shown in Figure 1. The maze frame consisted of a circular wooden center platform, 3.0 m in diameter, 11 vertical pillars equally spaced around the center platform (standing 2.3 m tall), and a circular ring around the top to stabilize the pillars. To create the walls of each arm, both ends of a solid black tarp were attached to sequential pillars at the edge of the center platform, and then wrapped around a heavy 2-m tall cylinder forming the ends of each arm. The complete maze had 11 equally spaced arms extending from the center area, each 5 m long by 1 m wide, resulting in a total maze width of 13 m. An 11-arm design was employed to create an asymmetrical arm pattern, thereby decreasing the chances for systematic strategies. This arrangement also allowed us to compare processing capacity of humans and rodents, which has classically been described by Miller as 7 ± 2 items of information in humans (Miller, 1956). More recent work has described working memory capacity limits of 3-5 pieces of information under certain circumstances, like those with abstract concepts (see: Cowan, 2010). However, human working memory capacity for more ecological tasks, such judging spatial locations in a radial-arm maze setting, has specifically been estimated as 7 ± 2 items of information (Glassman et al., 1994, 1998). Glassman et al. (1994, 1998) promoted RAMs as a means to test memory capacity by assuming that each maze arm represents one item of information, which allows a precise definition of capacity associated with repetition error rate. Use of such mathematical analysis principles facilitates determination of a behavioral metric of working memory capacity that is common to rodents and humans. A behavioral metric of capacity presumably includes both abstract core storage and related processing mechanisms such as proprioceptive recall of egocentric physical orientation or use of external room cues, and which allows a means of systematically testing such parameters. We built the maze with walls that extended above human height, and required participants to retrieve rewards from all 11 arms to complete the maze. This provided a fully translational RAM task with actual locomotive motor movements, and full realism, as compared to virtual reality versions that can produce distortions due to computer lag, and lack a fully realistic array of location and depth cues. On the floor at the end of each arm was a 2′ × 3′ (0.6 × 0.9 m) solid black floor mat, which served to conceal a reward. Each reward was a single bill of fake paper money; denominations varied across rewards. External visual cues on the room walls were present, including two basketball hoops on opposite ends of the room, solid black posters, and a clock. The maze was built in an Arizona State University gymnasium 57′ wide × 90′ long × 26′ tall (17.4 m wide × 27.4 m long × 7.9 m tall).

Instructions were given to introduce the participants to the goals of the task and to prevent participants from simply sequentially proceeding down successive arms or every other arm. This was done to encourage participants to use utilize spatial strategies or to utilize more complex strategies than simple chaining to traverse the maze. Each participant was read the following instructions prior to maze testing:

“Money is under the mat at the end of each arm of this maze. Your goal is to find all of the money in the shortest amount of time. Once you find the money in an arm, it will not be replaced. Therefore, you should avoid going into any arm twice. Do not enter arms that are immediately next to each other or go in a pattern entering every other arm. Only travel into an arm immediately next to the one you previously entered if you absolutely must in order to obtain the remaining money, which means only do it when you are almost certain that you are on your last reward. If you do travel into an arm immediately next to the previous one you will be asked to stop and return to the center. Once you find money, please return it to the researcher located in the center of the maze. Then, wait until they tell you to go, and proceed to the rest of the arms to collect the remaining money. During the course of testing please do not ask the researcher how many rewards remain or any other questions regarding your performance, as they are not permitted to respond.”

Each participant started at the center of the maze; after receiving the instructions, the participant was told to begin collecting the rewards from each arm. For each trial, the researcher recorded the exact arm(s) the participant went down and recorded the time it took the participant to discover each reward. Upon locating a reward, the participant was instructed to return to the center of the maze, hand the reward to the researcher and then continue on to the next trial. This process was repeated until all 11 rewards were located, resulting in 11 total test trials (testing session A).

Following successful collection of all 11 rewards, participants were brought outside the maze and administered the WRAT-3, which served as a general measure of verbal intelligence. During this time (approximately 5 min), a second experimenter replaced all 11 rewards in the HRAM. After the WRAT-3, participants were tested on the HRAM a second time (testing session B), adhering to the same set of directions. Participants were scored based upon the number of total incorrect arm entries they made (HRAM Errors). Because all of the arms contain rewards at the beginning of each testing session, errors solely consist of repeat arm entries within each testing session and all errors are considered to be working memory errors. After completion of the HRAM and WRAT-3, participants were taken to a separate room and administered a general survey and the remaining cognitive tests.

### Intelligence measure

Between HRAM testing sessions, the WRAT-3 Reading subtest was administered, serving as a general measure of verbal intelligence. The WRAT-3 relies on the participants' ability to read aloud a list of increasingly less common irregularly spelled words, and is useful as an estimate of verbal intelligence (Lezak et al., 2004).

### Neurocognitive tasks

#### Episodic memory

The Rey Auditory Verbal Learning Task (RAVLT) was used to assess episodic memory ability. In this task, participants must listen to and verbally recall words from a 15-item word list (List A) in 5 consecutive recall trials (Trials A1-A5; Total Words Learned). List A is then followed by recall of a distractor list (List B) in a single trial (Trial B1), and an immediate recall of List A (Trial A6; Retroactive Interference), which is often used as a measure of retroactive interference and short-term memory. After 20 min, delayed memory/long term memory recall is assessed in a single recall trial (Trial A7; Delayed Recall). Participants were scored on the number of words recalled correctly on each trial. Scores for Trials A1–A5 (Total Words Learned) were the total number of correctly recalled words across all five trials. Finally, participants complete a recognition trial discriminating words from List A from foils. We did not standardize scores by age or sex, but rather acknowledged age and sex as potential demographic variables that may influence scores on multiple tasks. Given that the rodent RAM has been reliably shown to be sensitive to both age and sex, this best facilitated our goal to examine the relationship between variance in our cognitive test scores and variance in our HRAM scores.

#### Visuospatial ability

Two paper-and pencil measures of visuospatial ability were used in this study. The first was a version of the Vandenberg and Kuse Mental Rotation Task (MRT) (Vandenberg and Kuse, 1978), redesigned by Peters et al. (1995). Version A of the MRT was used, which consists of 4 practice and 24 test questions. Each question is composed of five simple three-dimensional images made up of blocks. For each question, the objective is to match the target figure to a rotated version that is presented among a group of distractor items, which are either mirror images of the target figure or a different shape than the target figure. Participants were given 2 min to read the instructions and complete the practice items (not scored), 3 min to complete the first 12 items, and another 3 min to complete the remaining 12 items. Answers are considered correct only if the participant selects both correct images, with no partial credit for only one correct item (Peters et al., 1995). We also used the Judgment of Line Angle and Position-15 (JLAP) (Collaer, 2001) to measure visuospatial ability. The JLAP-15 consists of 20 test items and 5 practice items; each test item consists of two target line segments located directly above the comparison spectrum of 15 numbered lines in a 180° array. The target line segments were each 1 cm in length, whereas the comparison lines were 3 cm in length (Cherney and Collaer, 2005). Participants were given 2 min to read the instructions and complete the practice items (not scored), after which they were given 7 min to complete as many of the 20 test items as possible. Credit for correct answers is given only when both of the correct target lines are identified, with no partial credit for only one correct line. For the MRT and the JLAP, the score assigned was the total number of items answered correctly.

#### Working memory capacity

Working memory was assessed by a set of four computerized complex-span working memory tasks. These tests require participants to maintain mental memoranda (either verbal or spatial) in the face of completing a distracting task. These tests included verbal (Operation Span; OSpan and Reading Span; Rspan) and spatial (Symmetry Span; SymSpan and Rotation Span; RotSpan) working memory tasks (see Unsworth et al., 2009 for full task descriptions). In complex-span tasks, the participant is given verbal or spatial memoranda interspersed with distracting activity for a set of lists containing between 3 and 7 items. The participant's task is to remember the information in the order it was presented while simultaneously completing the distractor task. In all working memory tasks the dependent variable was the number of correct items recalled in the correct serial position.

### Task administration overview

The HRAM (testing session A) was the first task participants completed as a measure of spatial working memory. The WRAT-3 was administered between the two HRAM testing sessions as a measure of verbal intelligence. The WRAT-3 was followed by a second HRAM testing session (testing session B), to determine whether participants improved performance across testing sessions. After completion of the second session of HRAM testing, participants completed a survey regarding health and demographic factors. Participants were then administered the RAVLT Trials A1-A6, MRT, JLAP, RAVLT Trial A7, and computer tasks. The testing battery was given in the same order for all participants. Upon completion of all tasks, participants were debriefed. The total time from beginning to completion was approximately 2 h per participant.

### Statistical analyses

HRAM data were analyzed using repeated measures ANOVA, with HRAM Errors on Trials 1–11 and Sessions A and B as the repeated measures. Relations between performance on the MRT, JLAP-15, RAVLT, WRAT-3, RSpan, OSpan, SymSpan, and RotSpan with HRAM performance were examined with correlations and multiple regression analysis. In order to determine the extent to which each task predicts performance on the HRAM, a real-world, immersive task requiring spatial navigation, learning and memory, individual regressions were run with each task serving as the predictor and total errors made on both sessions of the HRAM combined (HRAM Total Errors) as the dependent (predicted) variable.

Additionally, hierarchical regression analysis was utilized to determine whether tasks measuring different domains of learning and memory offered unique predictive value to a regression equation predicting HRAM scores. Tasks that emerged as significant predictors of HRAM performance were entered into a regression equation in sequence, starting with the tasks that accounted for the largest proportion of variance in HRAM scores. Tasks were entered in clusters according to which cognitive domain they measure. The dependent variable for all equations was total errors made on both sessions of the HRAM combined (HRAM Total Errors). Four measures of working memory capacity, OSpan, RSpan, RotSpan, and SymSpan accounted for the largest proportion of variance in HRAM Total errors, and were entered as a first block of predictors to yield Equation (1):

(1) $$\begin{array}{l} \mathrm{HRAM}\;\mathrm{Total}\;\mathrm{Errors}=b_{1}\mathrm{OSpan}\;+\;b_{2}\mathrm{RSpan}\;+\;b_{3}\mathrm{RotSpan}\\ \;+b_{4}\mathrm{SymSpan}\;+\;b_{0} \end{array}$$

Two measures of visuospatial ability, MRT and JLAP were added to yield Equation (2):

(2) $$\begin{array}{l} \mathrm{HRAM}\;\mathrm{Total}\;\mathrm{Errors}=b_{1}\mathrm{OSpan}+b_{2}\mathrm{RSpan}+b_{3}\mathrm{RotSpan}\\ \;+b_{4}\mathrm{SymSpan}+b_{5}\mathrm{MRT}+b_{6}\mathrm{JLAP}+b_{0} \end{array}$$

Gain in prediction Equations (1) and (2) assessed prediction from visuospatial ability measures over and above working memory capacity^2^. Analyses were performed using SPSS 21 (IBM Corp. Released 2012. IBM SPSS Statistics for Windows, Version 21.0. Armonk, NY: IBM Corp.).

## Results

### Variable distributions and correlations

Means, standard deviations, and ranges for scores on our measured variables are presented in Table 1. Correlations among measured variables are presented in Table 2. The colors in the tables correspond with the colors in the figures, as associated with each group of tasks.

**Table 1:**
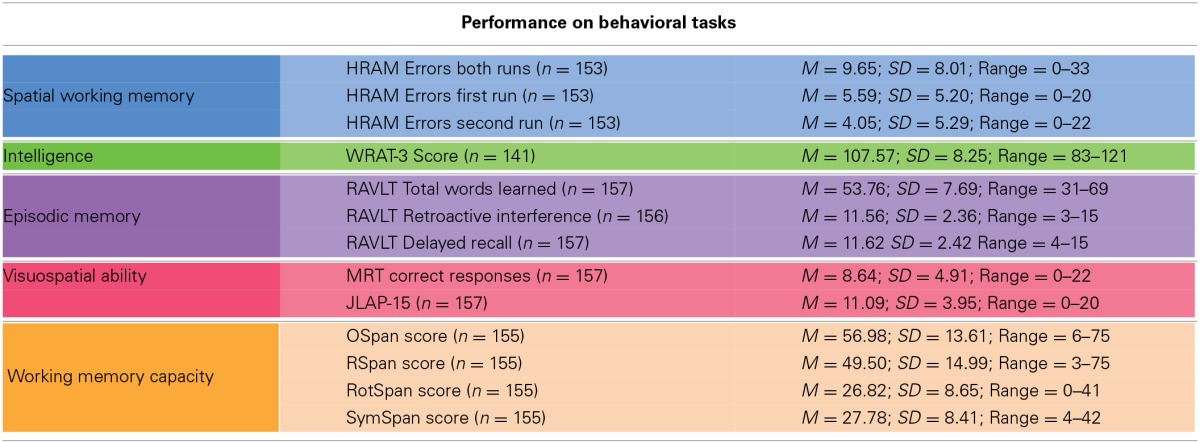
**Performance on behavioral tasks**.

*Mean, standard deviation, and range for each behavioral task*.

**Table 2:**
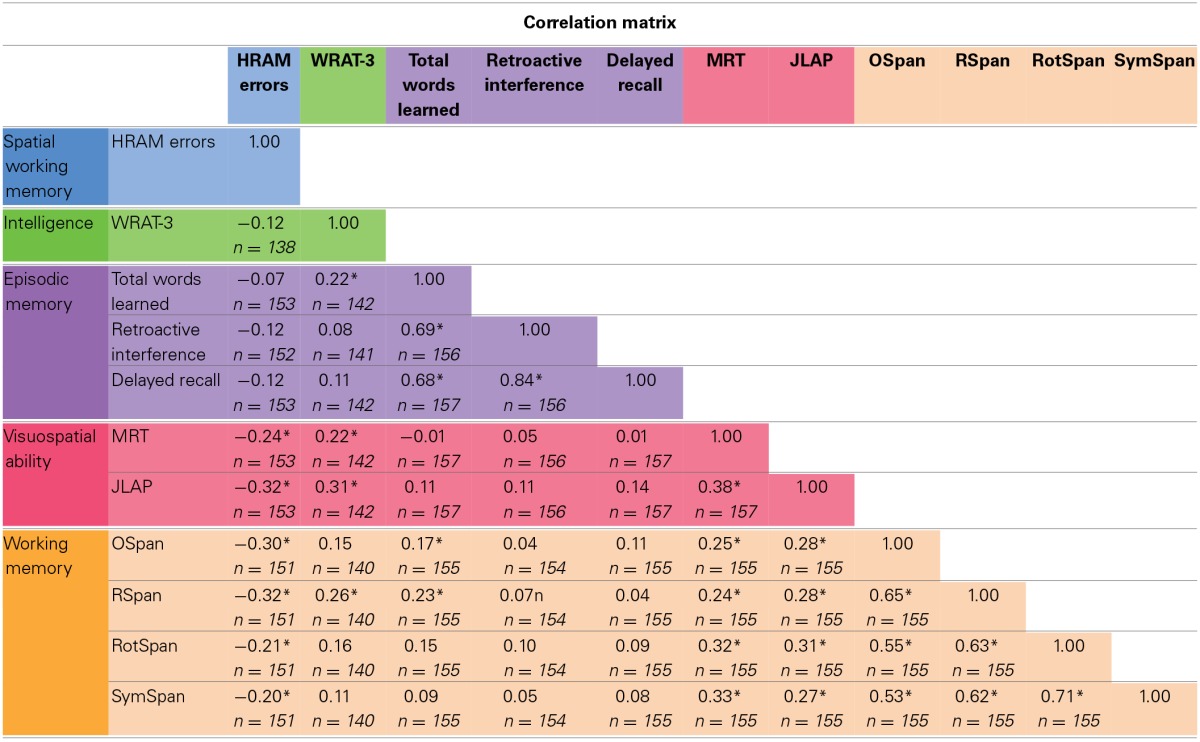
**Correlation matrix**.

Correlations and number of observations used for each behavioral task

^*^p < 0.05.

### Human radial arm maze performance

**Figure 2:**
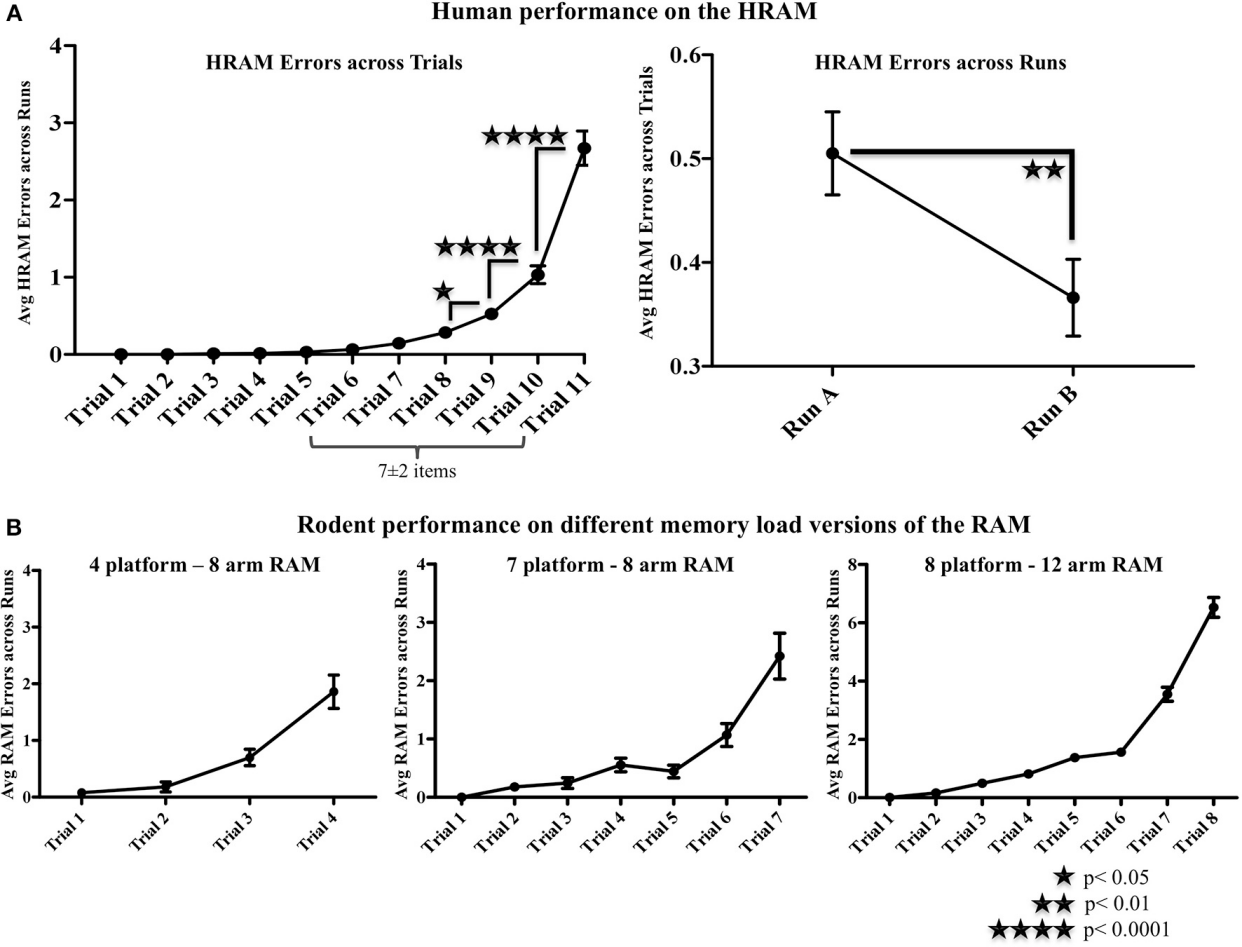
**Human and rodent radial arm maze performance. (A)** There was a significant main effect of Trial [*F*_(10, 1520)_ = 97.19; *p* < 0.0001] on HRAM Errors, with HRAM Errors increasing as trials progressed and working memory load increased. HRAM Errors increased from trial 8 to 9 (Trial 8: *M* = 0.28, *SE* = 0.04; Trial 9: *M* = 0.53, *SE* = 0.06; *p* < 0.05), from trial 9 to trial 10 (Trial 10: *M* = 1.03; *p* < 0.0001) and again from trial 10 to trial 11 (Trial 11: *M* = 2.67, *SE* = 0.11; *p* < 0.0001). HRAM Errors declined significantly across Testing Sessions [Both Sessions: *M* = 0.436, *SE* = 0.04, Session 1: *M* = 0.51, *SE* = 0.04, Session 2: *M* = 0.37, *SE* = 0.04; *F*_(1, 152)_ = 7.85; *p* < 0.01]. **(B)** Similar patterns of performance are seen in the 7/8 arm (unpublished observations from the Bimonte-Nelson lab), 4/8 arm (see also: Camp et al., 2012), and 8/12 arm (see also: Bimonte-Nelson et al., 2003) versions of the rodent water RAM.

There was a significant main effect of Trial [*F*_(10, 1520)_ = 97.19; *p* < 0.0001] on HRAM Errors, with HRAM Errors increasing as trials progressed and working memory load increased (Figure 2A). HRAM Errors increased from trial 8 to 9 (Trial 8: *M* = 0.28, *SE* = 0.04; Trial 9: *M* = 0.53, *SE* = 0.06; *p* < 0.05), from trial 9 to trial 10 (Trial 10: *M* = 1.03; *p* < 0.0001) and again from trial 10 to trial 11 (Trial 11: *M* = 2.67, *SE* = 0.11; *p* < 0.0001). This increase in errors occurred when the number of arms participants needed to avoid exceeded roughly 8-9 items. HRAM Errors declined significantly across Testing Sessions [Both Sessions: *M* = 0.436, *SE* = 0.04, Session 1: *M* = 0.51, *SE* = 0.04, Session 2: *M* = 0.37, *SE* = 0.04; *F*_(1, 152)_ = 7.85; *p* < 0.01]. The pattern of performance across trials was the same across Testing Session, [Session x Trial interaction: F_(10,1520)_ = 1.80; *p* > 0.05, NS]. Figure 2B shows error patterns observed in different versions of the rodent RAM for comparison.

### Relations between neurocognitive tasks and HRAM performance

#### General intelligence

**Figure 3:**
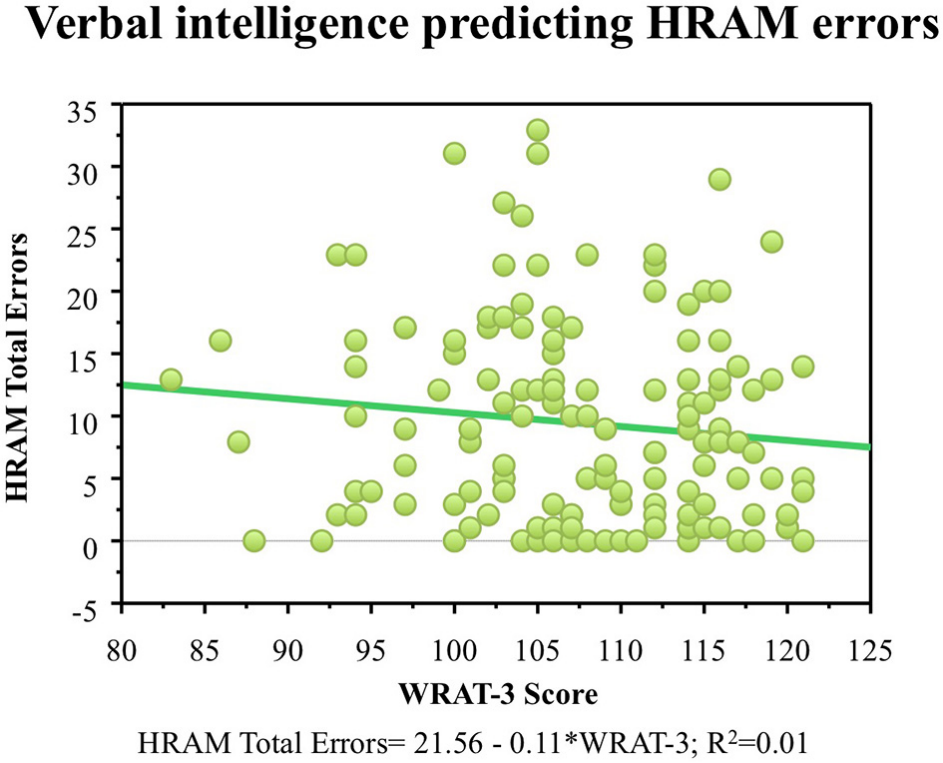
**HRAM scores as predicted by verbal intelligence measure**. Regression analysis indicated that the WRAT-3 was not a significant predictor of HRAM Total Errors [*F*_(1, 135)_ = 1.85, *p* > 0.05].

WRAT-3 scores did not correlate with HRAM Total Errors (Table 2). Consistent with the lack of correlation, the WRAT-3 was not a significant predictor of HRAM Total Errors (Table 3; Figure 3).

**Table 3:**
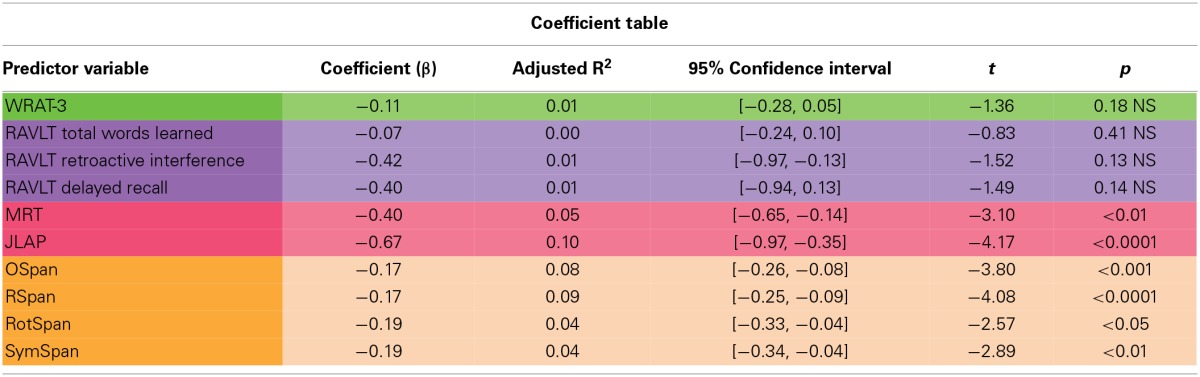
**Coefficient table**.

*Coefficients (β), adjusted R^2^, 95% confidence intervals, t-values and significance levels for each predictor of HRAM performance*.

#### Episodic memory

**Figure 4:**
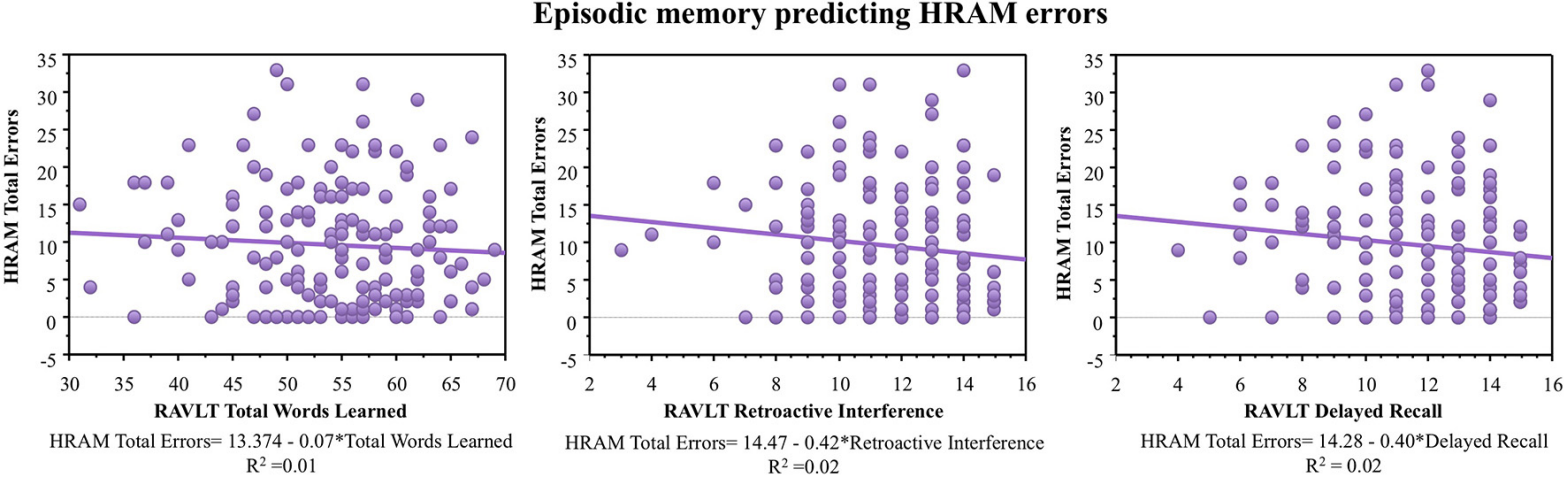
**HRAM scores as predicted by episodic memory measures**. Regression analysis indicated that Total Words Learned [*F*_(1, 151)_ = 0.69, *p* > 0.05], Retroactive Interference [*F*_(1, 150)_ = 2.31, *p* > 0.05], and Delayed Recall [*F*_(1, 151)_ = 2.23, *p* > 0.05] trials of the RAVLT were not significant predictors of HRAM Total Errors.

As shown in Table 2, RAVLT Total Words Learned, Retroactive Interference, and Delayed Recall did not correlate with HRAM Total Errors (*p* > 0.05, NS). Regression analysis indicated that Total Words Learned, Retroactive Interference, and Delayed Recall trials of the RAVLT were not significant predictors of HRAM Total Errors (Table 3; Figure 4). Combining all measures of the RAVLT also did not predict HRAM Total Errors [Adjusted R^2^_multiple_ = 0.00, *F*_(3, 151)_ = 0.88, *p* > 0.05, NS].

#### Visuospatial tasks

**Figure 5:**
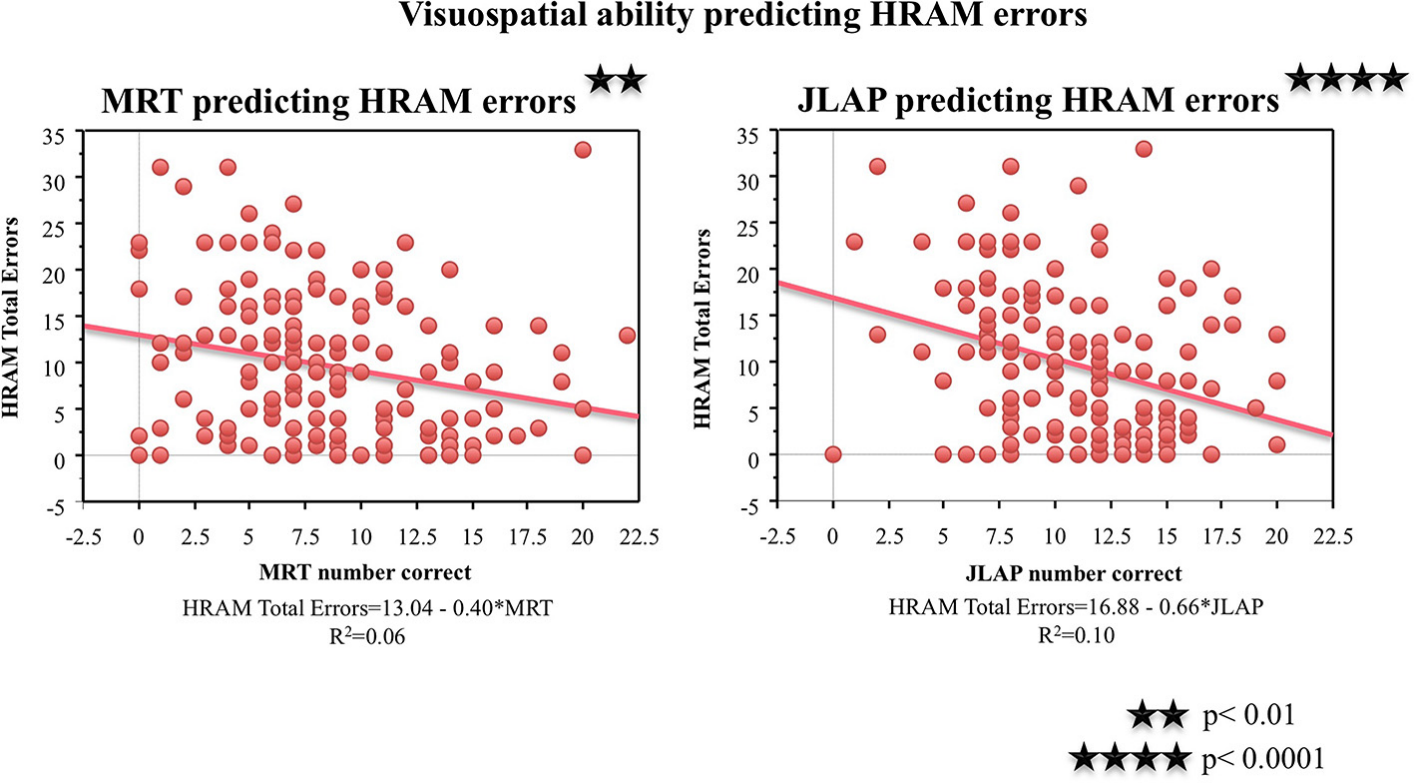
**HRAM scores as predicted by visuospatial ability measures**. Regression analysis indicated that MRT [*F*_(1, 151)_ = 9.63, *p* < 0.01] and JLAP [*F*_(1, 151)_ = 17.38, *p* < 0.0001, *R*^2^ = 0.66] performance predicted HRAM Total Errors.

As shown in Table 2, both visuospatial tasks, the MRT and JLAP, correlated negatively with HRAM Total Errors (*p* < 0.01 and *p* < 0.0001, respectively). For every additional question participants answered correctly on the MRT, HRAM errors decreased by 0.40 on average; errors decreased by 0.66 for each one point increase in JLAP (Table 3; Figure 5). The MRT and JLAP together predicted HRAM Total Errors [Adjusted R^2^_multiple_ = 0.11, *F*_(2, 152)_ = 10.29, *p* < 0.0001].

#### Working memory capacity tasks

**Figure 6:**
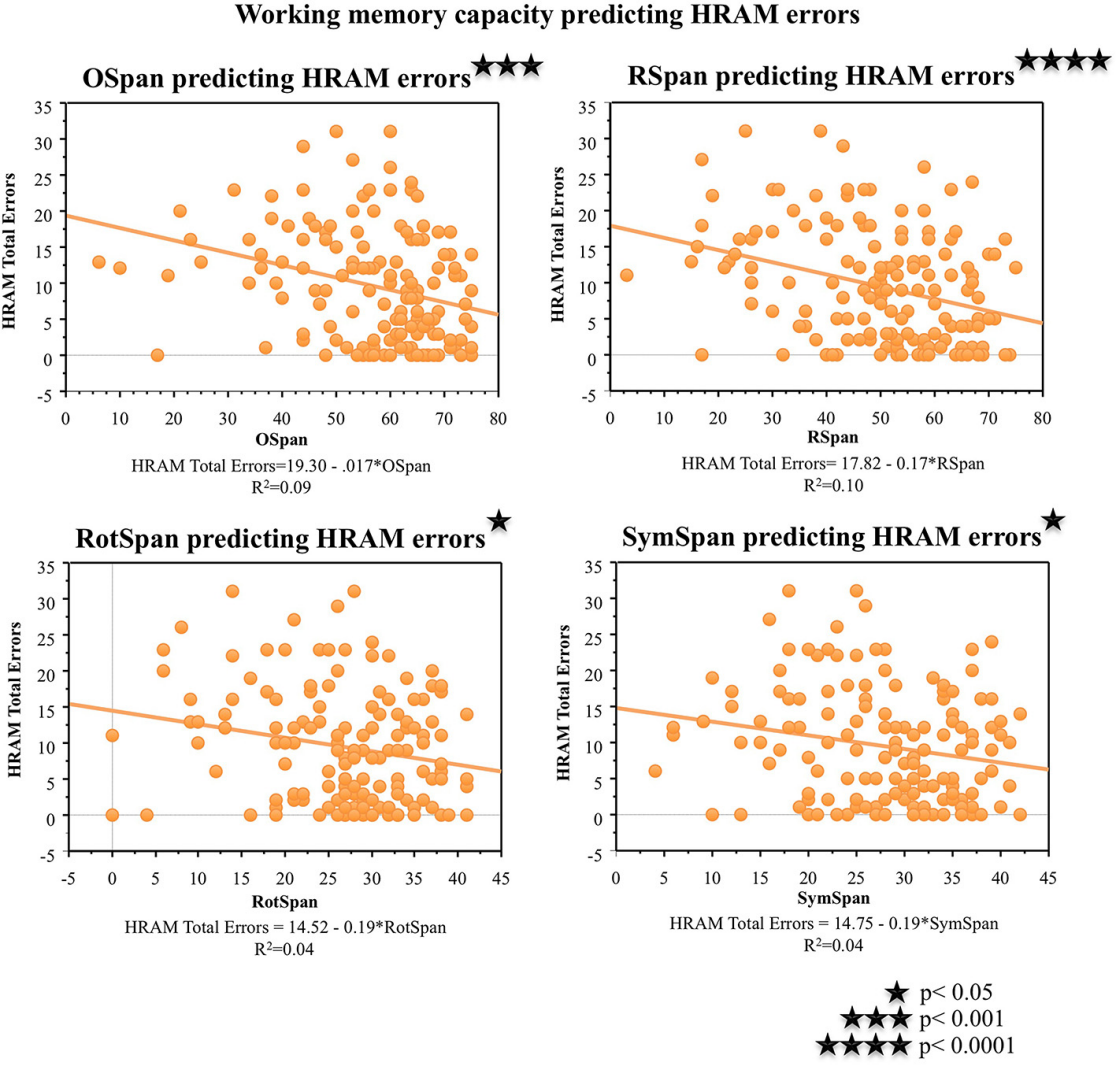
**HRAM scores as predicted by working memory measures**. Regression analysis indicated that Ospan [*F*_(1, 149)_ = 14.46, *p* < 0.001], RSpan [*F*_(1, 149)_ = 16.65, *p* < 0.0001], RotSpan [*F*_(1, 149)_ = 6.62, *p* < 0.05], and SymSpan [*F*_(1, 149)_ = 6.44, *p* < 0.05] performance predicted HRAM Total Errors.

As shown in Table 2, performance on the working memory capacity tasks, the OSpan, Rspan, RotSpan, and SymSpan, correlated negatively with HRAM Total Errors (*p* < 0.001, *p* < 0.0001, *p* < 0.05, *p* < 0.05, respectively). For every additional point earned on the Ospan or Rspan, HRAM Total Errors decreased by 0.17, on average; HRAM Total Errors decreased by 0.19 for each one point increase in RotSpan or SymSpan scores (Table 3; Figure 6). The Ospan, Rspan, RotSpan, and SymSpan together predicted HRAM Total Errors [Adjusted R^2^_multiple_ = 0.09, *F*_(4, 146)_ = 4.80, *p* < 0.001].

### Unique predictive value of tasks measuring different domains of cognition

The baseline regression equation Equation (1), including working memory capacity predictor variables, accounted for a significant proportion of variance in HRAM Total Errors [Table 4; Adjusted R^2^_multiple_ = 0.09, *F*_(4, 146)_ = 4.80, p < 0.001]. Only the Rspan predicted HRAM Total Errors when all other WM Span test scores were held constant [β = −0.12, 95% CI: (−0.24, 0.00), *t* = −1.99, *p* < 0.05]; none of the other WM Span tasks offered unique predictive value in a regression equation including all four tasks [Ospan: β = −0.10, 95% CI: (−0.22, 0.02), *t* = −1.59, *p* > 0.05; RotSpan β = 0.01, 95% CI: (−0.20, 0.23), *t* = 0.13, *p* > 0.05; SymSpan: β = 0.01, 95% CI: (−0.20, 0.22), *t* = 0.09, *p* > 0.05].

**Table 4:**
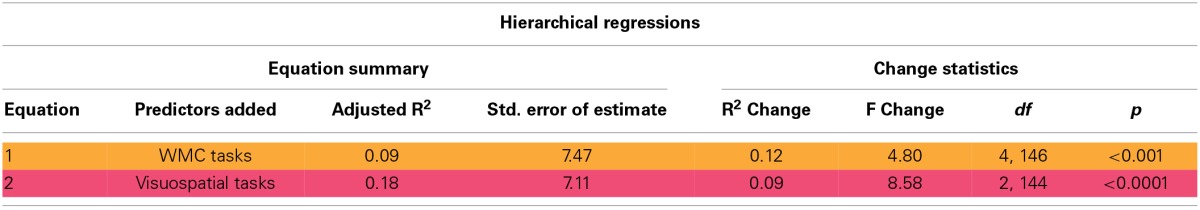
**Hierarchical regression summary**.

*Predictors, adjusted R^2^, standard error, R^2^ change, F change, degrees of freedom, and significance levels for each equation*.

The addition of two visuospatial tasks, MRT and JLAP, as predictor variables [MRT: β = −0.28; 95% CI (−0.55, −0.02); *t* = −2.14; *p* < 0.05; JLAP: β = −0.45; 95% CI (−0.78, −0.13); *t* = −2.75; *p* < 0.01] significantly increased the proportion of variance in HRAM Total Errors that was accounted for by our regression equation [Table 4; Adjusted R^2^_multiple_ = 0.18; *F*_change(2, 144)_ = 8.58; *p* < 0.0001]. The Adjusted R^2^_multiple_ for Equation 2 indicated that adding MRT and JLAP as predictors roughly doubled the proportion of explained variance in HRAM Total Errors. JLAP scores offered predictive value over and above MRT scores [β = −0.55, 95% CI: (−0.88, −0.21), *t* = −3.22, *p* < 0.01]; however MRT scores were not predictive of HRAM Total Errors when JLAP scores were held constant [β = −0.23, 95% CI: (−0.50, 0.03), *t* = −1.73, *p* > 0.05].

## Discussion

The current study employed a human-sized, walk-through version of the RAM that was modeled after the rodent version used commonly in learning and memory research. The RAM has been used for decades to study spatial memory in the rodent. Notable landmark work includes that of Tolman in the 1940s utilizing the structurally-similar sunburst maze (Tolman, 1948), Olton utilizing the RAM in the 1970s (Olton and Samuelson, 1976; Olton, 1977; Olton and Papas, 1979; Olton and Feustle, 1981), and more recent work many decades later (e.g., Eckerman et al., 1980; Luine and Rodriguez, 1994; Bimonte and Denenberg, 1999, 2000; Bimonte et al., 2000; Bimonte-Nelson et al., 2003, 2006; Daniel et al., 2006). Despite the many advantages of using animal models in research, there remain questions about the extent that findings in animals can truly be translated to humans, especially in the context of neurobehavioral assays. One approach to addressing this obviously complex issue is to create an intermediate testing instrument by adapting experimental paradigms from animals to humans. The present study did this through the development of the HRAM. Previous research teams have developed human versions of mazes, in particular the RAM, with their own unique set of parameters designed to answer their research questions (O'Connor and Glassman, 1993; Glassman et al., 1998; Bohbot et al., 2002; Scharine and McBeath, 2002; Astur et al., 2004; Levy et al., 2005). We built our version of the HRAM with these prior studies in mind, and optimized the parameters to be as comparable as possible to the rodent version. We expected to see an increase in working memory errors as trials progressed. As predicted, participants began to make errors around trial 6, with the highest number of errors made on trial 11, when working memory demand was at its highest (Figure 2A). The increase in errors across trials in the HRAM is similar to that shown in the RAM with rat subjects (Bimonte and Denenberg, 1999, 2000; Bimonte et al., 2000, 2003; Bimonte-Nelson et al., 2003; Camp et al., 2012), as seen in Figure 2B. Additionally, performance improved across testing sessions, indicating a learning effect, as seen in rodent RAMs (Bimonte and Denenberg, 1999, 2000; Bimonte et al., 2000, 2003; Bimonte-Nelson et al., 2003; Camp et al., 2012).

One major goal of this study was to explore the translational relationship between human performance on the HRAM and commonly used neuropsychological tests that tap spatial ability, episodic memory, working memory, and intelligence. Evaluating these relationships allowed us to determine which tests commonly used in clinical settings and cognitive psychology account for variance in performance on the HRAM, a commonly used rodent task adapted to humans. Of the battery of tests we administered in this study, the JLAP emerged as the strongest predictor of HRAM performance, accounting for 10% of HRAM Total Errors. The verbal working memory capacity tasks, the Ospan and Rspan, surfaced as the next strongest predictors, predicting 8 and 9% of the total variance in HRAM error scores, respectively. The MRT predicted 5% of variance on HRAM Total Errors, and the predictive value of the spatial working memory tasks (the RotSpan and SymSpan) was similar to the predictive value of the MRT, each predicting 4% of the total variance in HRAM error scores (Table 3). Total Words Learned, Retroactive Interference, and Delayed Recall measures of the RAVLT did not offer significant predictive value, nor did scores on the WRAT-3, an estimate of general intelligence.

When evaluating the nature of these tasks, plausible explanations for the observed relationships emerge. The predictive ability of the MRT and JLAP-15 may be attributable to the proposed use of a mental visuospatial sketchpad (Baddeley, 2000). The visuospatial sketchpad has been theorized to be the temporary storage and manipulation of spatial and visual information, such as shapes, locations or speed of objects in space. The visuospatial sketchpad is theorized to contribute to performance in tasks that require planning of spatial movements, such as planning one's way through a complex environment like the HRAM and it is not surprising that better performance on visuospatial tasks predicts enhanced performance in a three-dimensional, immersive task that requires navigation through space. The working memory tasks used in this study assess the ability to “hold on” to multiple pieces of information in the face of interference and an increase in working memory demand (e.g., Baddeley, 2003; Unsworth et al., 2009). Similarly, to perform well on the HRAM, participants must also maintain multiple pieces of information in the face of an increasing working memory load to successfully complete the task.

Performance on the HRAM did not correlate with the estimate of general verbal intelligence used in this study (reading subtest of the WRAT-3) or with new learning and long term delay measures of episodic memory, but did correlate with specific measures of visuospatial ability and working memory capacity, suggesting that the HRAM requires utilization of specific cognitive abilities of working memory and visuospatial skills rather than reliance on episodic memory or general verbal intelligence, as measured by the WRAT-3. Thus, our findings indicate that tasks measuring working memory (e.g., maintaining performance within the context of increased load or distracting stimuli) and visuospatial skills are correlated with performance on the RAM, a task used widely in rodent literature that we have fully adapted to human proportions.

Hierarchical regression analysis indicated that the proportion of HRAM error variance accounted for by each group of predictor variables (working memory capacity and visuospatial ability) was unique to that group of variables. Scores on the MRT and JLAP accounted for 9% of variance in HRAM scores, in addition to the 9% of variance accounted for by the four working memory capacity variables (Table 4). A regression equation including the working memory capacity tests and visuospatial ability tests, accounted for 18% of the total variance in HRAM error scores. These results suggest that including multiple measures in a cognitive battery increases the ability of the battery to predict how a participant would perform on tasks similar to the HRAM, which requires complex reasoning, such as recall of previous instances of navigating to spatial locations in a real-world setting. Additionally, these results support the hypothesis that successful performance on radial-arm maze tasks requires visuospatial abilities and sufficient working memory capacity.

In conclusion, our collaborative research group created a three-dimensional, fully immersive, walk-through version of the RAM designed specifically for human use, in order to create an intermediary translational instrument. The results indicate that human performance on the HRAM is notably similar to rodent performance on the RAM, in that there is an exponential increase in errors as trials progress and task difficulty increases, but with the human error pattern revealing a larger processing capacity compared to rodents. The total number of errors per trial in humans remains low until the trial number exceeds a total similar to the classically defined human working memory capacity of 7 ± 2 items (Miller, 1956). Additionally, HRAM performance in our participants improved with repeated exposure to the task, indicating learning. We also demonstrated that performance on the HRAM was related to performance on several tasks used in clinical neuropsychology and cognitive psychology, with a strong emphasis on tasks designed to measure spatial ability and working memory. The behavioral similarities seen in the rodent and human versions of the RAM, paired with the strong observed relationships between the HRAM and standard human working memory and visuospatial tasks, offer support to spatial working memory being the dominant construct common to rodents and humans that is reliably measured using existing testing procedures. Moreover, the HRAM has now been validated as a valuable instrument to translate, compare, and confirm models and findings in rodent research, cognitive neuroscience, navigational modeling, and neuropsychology. We took a collaborative and translational approach to bridge gaps between divergent, but closely related, fields of experimental and applied memory research. The successful implementation of the HRAM confirms our overarching goal to create a practical and useful basic—to applied- translational test instrument that can help us connect diverse behavioral domains to better understand learning, memory, and cognitive functioning processes.

### Conflict of interest statement

The authors declare that the research was conducted in the absence of any commercial or financial relationships that could be construed as a potential conflict of interest.

## Footnotes

^1^Three participants were omitted from analyses due to missing data on one or more predictors. Four additional participants were omitted from analyses due to unusual scores on the HRAM tasks; HRAM Total Error scores for these participants were outside of two standard deviations of the mean. Thirteen participants were excluded from analyses of WRAT-3 Reading subtest performance because English was their second language, and two others were excluded due to abnormally low scores on the WRAT-3. All missing data were handled using listwise deletion.

^2^Change statistics are based on the unadjusted R 2 values
